# Differential regulatory role of AU-rich and GU-rich elements in *Trypanosoma brucei*

**DOI:** 10.3389/fmicb.2025.1724550

**Published:** 2026-01-23

**Authors:** Xuemin Guo, Wei-Wei Yang, Shinuan Zeng, Sha Yue, Liang Zhou, Shuru Zhou, Xiaobin Meng, Logen Liu

**Affiliations:** 1 Meizhou People’s Hospital, Meizhou, China; 2 Guangdong Engineering Technological Research Center for Clinical Molecular Diagnosis and Antibody Drugs, Meizhou, China; 3 Zhongshan School of Medicine, Sun Yat-sen University, Guangzhou, China; 4 Department of Gastroenterology, The Second Affiliated Hospital, University of South China, Hengyang, China; 5 Department of Surgery, LKS Faculty of Medicine, School of Clinical Medicine, HKU-SZH, The University of Hong Kong, Hong Kong, China; 6 School of Life Sciences, Guangzhou University, Guangzhou, China; 7 The Aoyang Cancer Institute, The Affiliated Aoyang Hospital of Jiangsu University, Zhangjiagang, China; 8 Clinical Research Center, The Second Affiliated Hospital, University of South China, Hengyang, China; 9 Hunan Provincial Key Laboratory of Basic and Clinical Pharmacological Research for Gastroenterological Tumors, Hengyang, China

**Keywords:** AU-rich element, GU-rich element, post-transcriptional regulation, RNA-bindingprotein, *Trypanosoma brucei*

## Abstract

Post-transcriptional regulation is the predominant mode of gene expression control in *Trypanosoma brucei*, yet the underlying regulatory elements and proteins remain poorly defined. AU- and GU-rich elements (AREs and GREs) are common post-transcriptional regulatory motifs. To investigate their roles in *T. brucei*, we analyzed transcriptomic datasets and extracted 5,840 genes with defined 5′ and 3′ untranslated regions (UTRs), including 327 that are developmentally regulated between the parasite’s two life stages. Computational analysis revealed that AU- and GU-rich elements are widespread and enriched in the 3′UTRs of developmentally regulated mRNAs as well as in transcripts with long half-lives. Functional assays demonstrated regulatory activity of AREs and GREs within the 3′UTRs of five representative genes (*ICP*, *TOP2*, *MCC*-β, *PK*, and *KREPB6*), with differential effects on reporter expression. Notably, the GREs in the *ICP* and *TOP2* 3′UTRs destabilized reporter transcripts in procyclic-form trypanosomes but enhanced expression in bloodstream forms. RNA pulldown assays further identified DRBD2 as a potential GRE-binding protein, and DRBD2 knockdown reduced *ICP* mRNA abundance in procyclic trypanosomes. Collectively, these findings demonstrate that AREs and GREs are critical regulatory elements in *T. brucei*, exhibiting gene-specific and context-dependent functions. Elucidating their regulatory roles and identifying additional binding proteins will provide new insights into the mechanisms of post-transcriptional control in this parasite.

## Introduction

*Trypanosoma brucei*, a unicellular kinetoplastid protozoan, causes African sleeping sickness in humans and nagana in animals. *T. brucei* infection is transmitted by an insect vector, the tsetse fly (Büscher et al., 2017; Li et al., 2017). The proliferating stages of *T. brucei* in mammals and tsetse flies are bloodstream forms (BFs) and procyclic forms (PFs), respectively (Clayton, 2019). To adapt to completely different living environments, *T. brucei* developmentally expresses a large number of genes, ranging from 5 to 30% of its total genes, as revealed by microarray assays and high-throughput RNA sequencing (Briggs et al., 2023; Jensen et al., 2009; Kolev et al., 2010; Naguleswaran et al., 2018; Naguleswaran et al., 2021; Nilsson et al., 2010; Savage et al., 2016; Siegel et al., 2010). Gene expression in trypanosomes is believed to be mainly regulated at the post-transcriptional level, due to polycistronic transcription of functionary unrelated genes (Clayton, 2019; Singh et al., 2025). Although interactions between trans-acting RNA-binding proteins (RBPs) and cis-acting regulatory elements are central to post-transcriptional regulation, they remain largely uncharacterized in *T. brucei* (Clayton, 2019; Singh et al., 2025).

Several groups of protein domains recognized for RNA binding have been characterized, including the RNA recognition motif (RRM), the zinc finger domain, the acetylation lowers binding affinity (ALBA) domain, and the Pumilio domain (Kolev et al., 2014). A single protein overexpression of the RRM containing protein RBP6 in procyclic form *T. brucei* triggers differentiation to metacyclic trypomastigotes, which express the variant surface glycoprotein (VSG) coat (Kolev et al., 2012). Expression of RBP10 promotes the bloodstream form differentiation as well (Mugo and Clayton, 2017; Wurst et al., 2012). Both proteins exhibit a binding preference toward AREs (Mugo and Clayton, 2017; Najafabadi et al., 2013). Double RNA binding domain protein 18 (DRBD18) plays a key role in modulating transcript poly(A) site selection, stability and translation, thereby shaping life cycle specific 3′UTR isoforms (Bard et al., 2024; Bishola Tshitenge and Clayton, 2022; Ciganda et al., 2023). CCCH zinc finger containing ZC3H20 and ZC3H21 was reported to stabilize cytosolic and mitochondrial RNAs in procyclic *T. brucei* (Ling et al., 2011; Liu et al., 2020). Strikingly, knockout of ZC3H20 in trypanosomes resulted in a failure to differentiate toward stumpy bloodstream forms (Briggs et al., 2021). ZC3H22 suppresses the expression of genes involved in cell growth and proliferation while concomitantly enhancing the expression of several epimastigote associated markers (Erben et al., 2021). The ALBA proteins are primarily cytoplasmic proteins, ALBA1/ALBA2 interacting with GPEET procyclin 3′UTR (Mani et al., 2011; Subota et al., 2011). ALBA3 is associated with the translational machine and is required for the differentiation of mammalian stumpy forms into procyclic forms (Bevkal et al., 2021). Additional regulators such as PUF9, which stabilizes S-phase mRNAs via the UUGUACC motif (Archer et al., 2009), further enrich the regulatory network.

Early works in *T. brucei* established that cis-regulatory elements within 3′UTRs exert powerful effects on mRNA stability and translation. Studies on the EP and GPEET procyclin transcripts identified several discrete regulatory motifs, including the 16-mer and 26-nt elements within the *EP* 3′UTRs (Hehl et al., 1994; Irmer and Clayton, 2001) and elements within the *GPEET* 3′UTR (Furger et al., 1997). A non-coding RNA encoded between the two procyclin genes in the genomic region, *TblncRNA-23*, was also reported to regulated they expression via base paring to their 3′UTR region (Galili-Kostin et al., 2025). Besides to gene-based methods, genome-wide approaches via bioinformatic, reporter gene library coupled to next generation sequencing, and single cell sequencing also found sets of regulatory sequences (Gazestani et al., 2014; Trenaman et al., 2024). AREs were found among a set of linear and structural cis-elements within 3′UTRs that can modulate transcript abundance in a stage-dependent or condition-specific manner (Najafabadi et al., 2013). An adenine-rich poly-purine tracts was found to enhance translation, among thousands of regulatory sequences (Trenaman et al., 2024).

AREs, one of the most common regulatory signals in eukaryotic genes, are characterized by AUUUA pentamer repeats located within A- or U-rich regions (Khabar, 2017). Since AREs were first identified in the 3′UTRs of the genes encoding tumor necrosis factor (TNF) and some other cytokines in humans, the list of ARE-containing genes has expanded quickly. Approximately 7% of human protein-coding genes have been estimated to contain AREs (Bakheet et al., 2018). AREs are generally regarded as destabilizing elements; however, their regulatory effects depend on the specific proteins they recruit. For example, tristetraprolin and AUF1 primarily promote mRNA degradation upon binding to AREs, whereas HuR enhances mRNA stability (Lee et al., 2025; Ma et al., 2025; Zhang et al., 2021). In addition to their role in mRNA stability, AREs are also involved in the control of mRNA translation and export (Kattan et al., 2023; Otsuka et al., 2019). Previous studies have indicated that AREs are abundant in *T. brucei* (Najafabadi et al., 2013). Although they have been proposed to act as important regulatory elements, the functions of AREs in trypanosomes have not been well characterized. In *T. brucei*, several ARE-binding proteins have been identified; these include three ELAV-like homologs (DRBD13, TbRBP6, TbRBP10, and DRBD12) and a zinc finger protein, ZC3H11 (Droll et al., 2013; Jha et al., 2015; Najafabadi et al., 2013). Given the abundance of AREs in this parasite, additional ARE-binding proteins are likely to be identified, which will further advance our understanding of post-transcriptional regulatory mechanisms.

GU-rich elements (GREs) share a sequence pattern similar to that of AREs but have received relatively fewer attention. A genome-wide search of human genome showed that at least 5% of human transcripts contain GREs (Halees et al., 2011), comparable to the 7% abundance of AREs in human transcripts. Evidence from previous studies suggests that GREs regulate gene expression by modulating mRNA stability, translation, and pre-mRNA processing, largely through interaction with the Elav-like protein CELF1, which is also known as CUGBP1 (Nikonova et al., 2024; Zeng et al., 2025). Little is known about the abundance or function of GREs and GRE-binding proteins in trypanosomatid. Given that GREs are highly evolutionarily conserved and have been implicated in developmental processes across species ranging from worms to mammals (Nikonova et al., 2024; Qin et al., 2024; Sankaranarayanan et al., 2023; Vlasova-St Louis and Bohjanen, 2011), we hypothesize that GREs are likewise widespread in *T. brucei* transcripts and contribute to gene expression regulation in this organism.

In this study, we computationally analyzed the distribution of AREs and GREs in *T. brucei* using multiple published transcriptome datasets, revealing that these elements are abundant and enriched in the 3′UTRs of developmentally regulated mRNAs. Reporter assays confirmed the regulatory activity of AREs and GREs in several randomly selected genes. Importantly, a combination of biochemical and genetic approaches identified DRBD2 as a potential GRE-binding protein.

## Results

### AREs and GREs are abundant and are enriched in developmentally regulated genes

The transcriptome-wide distributions of AREs and GREs in *T. brucei* were examined. A total of 5,840 *T. brucei* gene transcripts for which both the 5′UTRs and 3′UTRs have been identified were obtained from published data (Supplementary Table 1; Jensen et al., 2009; Nilsson et al., 2010). AREs and GREs were searched throughout the UTRs or CDS, and the related genes were classified into five clusters based on the number of repeats of the core AUUUA or GUUUG sequence, with cluster I containing 5 repeats and cluster V containing only one. As shown in Table 1, 18.5 and 24% of the analyzed genes contain ARE or GRE, respectively, within their 3′UTRs. In contrast, a relatively smaller number of genes contain ARE or GRE within the 5′UTR or CDS. Notably, the number of genes containing cluster V ARE or GRE is much higher than the number of genes containing ARE or GRE belonging to other clusters. These results indicate that AREs and GREs, especially those belonging to cluster V, are highly abundant in the 3′UTRs of *T. brucei* genes. Further analysis of AREs and GREs across 3′UTRs revealed no positional bias (Supplementary Figure 1). Gene ontology (GO) analysis of the ARE- or GRE-containing genes revealed that GREs are mainly enriched in the genes related with mRNA stabilization, nucleotide phosphate biosynthetic process, and cytidine to uridine editing (Supplementary Figure 2).

**TABLE 1:** Classification and abundance of ARE and GRE in *T. brucei* genes.^a^

ARE/GRE motif sequence	Cluster	5′ UTR	CDS	3′ UTR
(AUUU)_5_A	I	0.38%	0.00%	0.74%
(AUUU)_4_A	II	1.13%	0.02%	1.54%
(AUUU)_3_A	III	2.47%	0.26%	3.70%
WWAUUUAUUUAWW	IV	0.70%	0.10%	0.98%
WWWWAUUUAWWWW	V	8.22%	2.98%	11.51%
(GUUU)_5_G	I	0.51%	0.09%	0.79%
(GUUU)_4_G	II	0.63%	0.00%	1.47%
(GUUU)_3_G	III	1.88%	0.45%	3.37%
KKGUUUGUUUGKK	IV	0.39%	0.51%	0.96%
KKKKGUUUGKKKK	V	9.42%	12.31%	17.57%

*^a^*For Cluster I to Cluster III, a single mismatch was allowed in the whole region. For Cluster IV and Cluster V, a single mismatch was allowed in flanking regions (i.e., the position of W or K). W represents A or U. K represents G or U.

**FIGURE 1:**
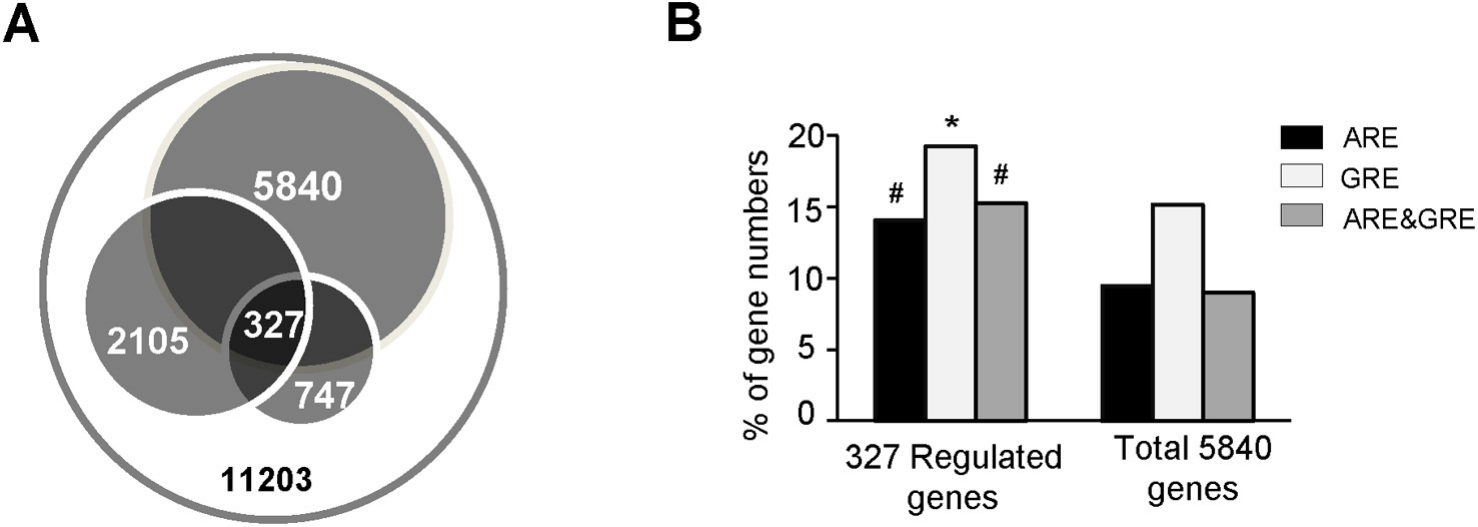
Distribution of genes containing AREs and/or GREs within 3′UTRs. **(A)** Schematic representation of regulated genes with defined 5′UTRs and 3′UTRs based on published datasets. A total of 11,203 genes are predicted in TriTrypDB. Among these, 5,840 genes possess both 5′ and 3′UTRs; 2,105 genes are developmentally regulated during the *T. brucei* life cycle (Jensen et al., 2009), and 747 genes are differentially expressed between cultured PFs and BFs stages (Siegel et al., 2010). The intersection of these datasets, comprising 327 genes, represents developmentally regulated genes with fully defined 5′ and 3′UTRs. **(B)** Comparison of the prevalence of genes containing AREs, GREs, or both (ARE&GRE) within their 3′UTRs between the total set of 5,840 genes and the 327 developmentally regulated genes. ^#^*P* < 0.01; *P < 0.05 compared with the total gene set (hypergeometric test).

To assess whether ARE- or GRE-containing genes are likely to be developmentally regulated, we compared their prevalence among all genes versus developmentally regulated genes. A total of 327 genes with annotated 5′ and 3′UTRs were reported in two published high-throughput studies as differentially expressed across life stages of *T. brucei* (Jensen et al., 2009; Nilsson et al., 2010; Figure 1A; Supplementary Table 1). Among these, 46, 63, and 50 genes contain AREs, GREs, or both (ARE&GRE), respectively, within their 3′UTRs, representing 14, 19.3, and 15.3% of the developmentally regulated gene set (Figure 1B). In contrast, ARE-, GRE-, and ARE&GRE-containing genes accounted for approximately 9.5, 15.2, and 9%, respectively, of the 5,840 total genes (Figure 1B). These results indicate that AREs and GREs are enriched in developmentally regulated genes, suggesting a role for these elements in developmental gene regulation.

### AREs and GREs are enriched in genes with long half-lives

**FIGURE 2:**
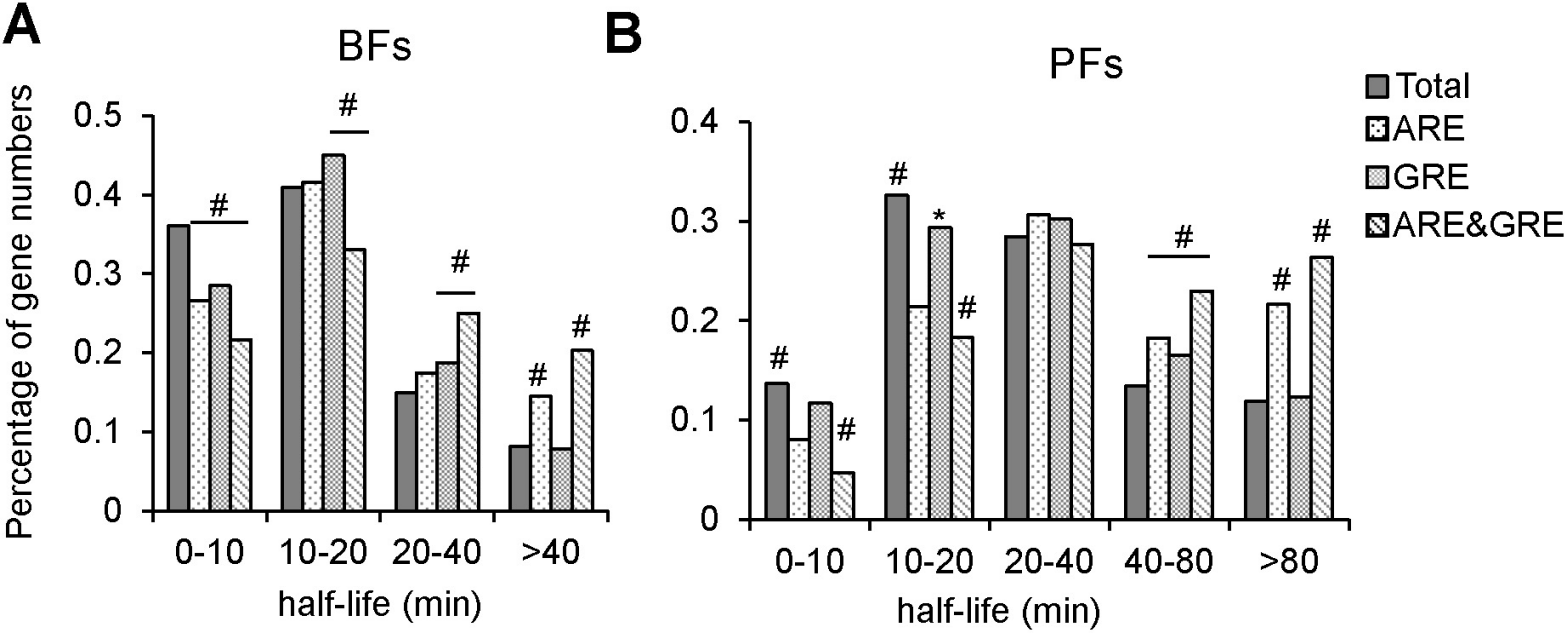
AREs and GREs are enriched in transcripts with long half-lives. Distribution of half-lives for ARE- and/or GRE-containing transcripts compared to all measured genes in BFs **(A)** and PFs **(B)**. Half-life data were obtained from Fadda et al. (2014). Statistical significance was determined using the hypergeometric test: ^#^*P* < 0.01, *P < 0.05 versus the total gene set with measured half-lives.

We analyzed the half-lives of genes containing AREs or GREs within their 3′UTRs. In *T. brucei*, the half-lives of 4,853 and 4,558 of 5,840 candidate genes have been determined in BF and PF, respectively (Supplementary Table 1; Fadda et al., 2014). Among these, 436, 758, and 448 genes in BF contain AREs, GREs, and both (ARE and GRE), respectively; in PF, the corresponding numbers are 411, 692, and 383. Genes were divided into four groups based on their half-lives ( < 10, 10–20, 20–40, and > 40 min), and the proportions of genes in each group were calculated and compared. Compared to the total gene set, ARE- or GRE-containing genes tend to produce more stable transcripts, with a smaller proportion having half-lives under 10 min and a larger proportion exceeding 20 min in BFs trypanosomes (Figure 2A). Notably, transcripts of genes containing AREs or ARE&GREs constitute a higher fraction of long-lived transcripts ( > 40 min). Similar trends were observed in PFs (Figure 2B). These results indicate that AREs and GREs are enriched in developmentally regulated genes with long-lived transcripts and that AREs and GREs are differentially associated with transcripts of varying stability.

### Gene- and life stage-specific regulatory effects of AREs and GREs in *T. brucei*

**FIGURE 3:**
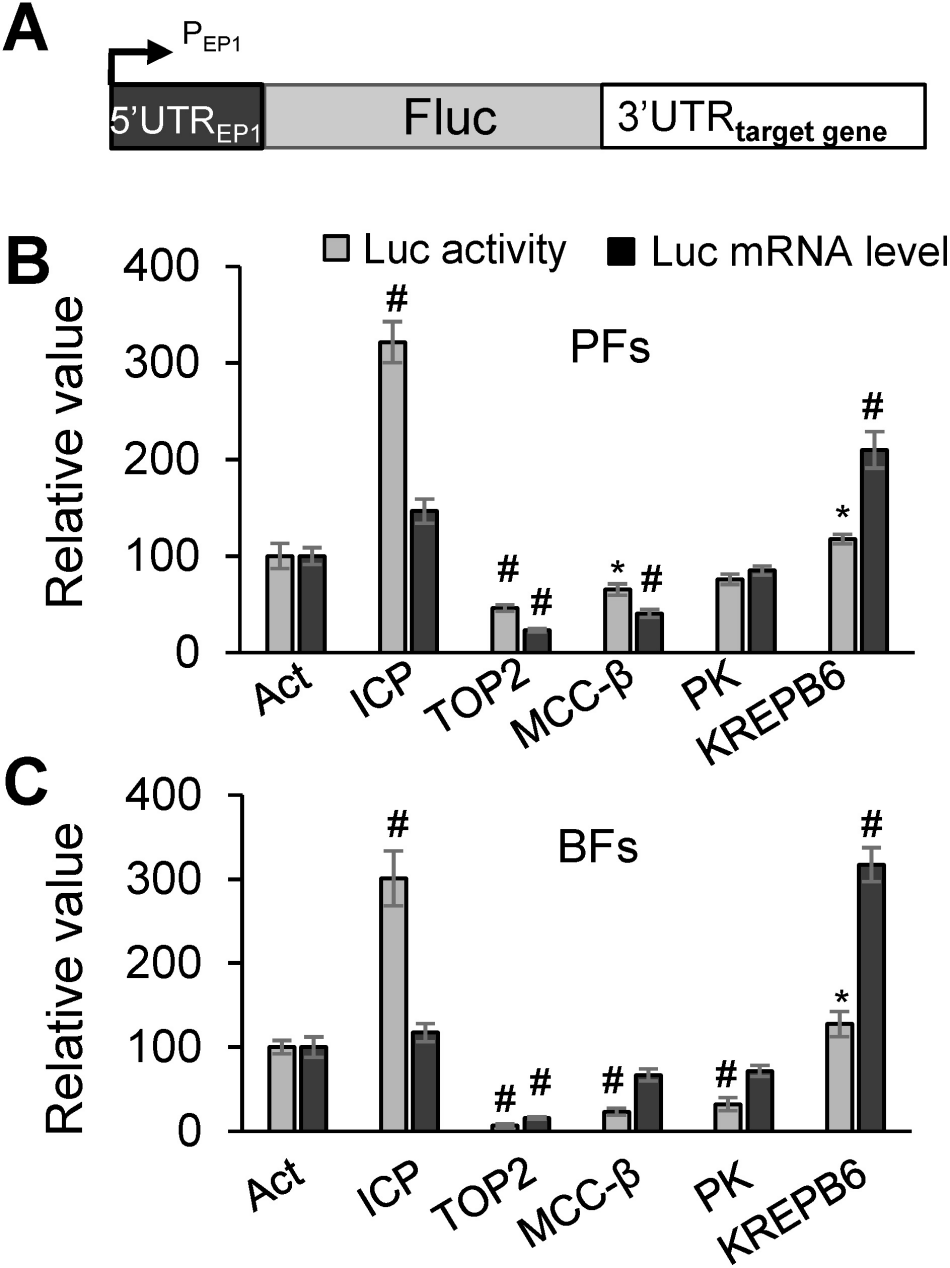
Reporter assay of the regulatory roles of 3′UTRs from selected genes. **(A)** Schematic of the luciferase reporter construct, including the promoter and the 5′ and 3′UTRs. **(B,C)** Effects of different 3′UTRs on reporter luciferase activity and mRNA steady-state levels in PFs **(B)** and BFs **(C)**. Reporter activity and mRNA levels for the construct containing the *actin A* (Act) 3′UTR were set to 100. Luc activity, relative luciferase value; Luc-3′UTR mRNA level, relative mRNA level determined by qRT-PCR compared to the *actin A* 3′UTR construct. At least three independent measurements were performed for each phleomycin resistant population, and error bars represent the standard error of the mean. Statistical significance: ^#^*P* < 0.01; *P < 0.05 versus the Act construct.

The regulatory roles of 3′UTRs containing AREs and/or GREs in gene expression were assessed using a reporter assay. The 3′UTRs of five ARE- and/or GRE-containing genes, i.e., *ICP*, *TOP2*, *MCC*-β, *PK*, and *KREPB6*, were cloned downstream of *Fluc* in the pLew79 vector, where FLuc expression was tetracycline (tet)-inducible (Figure 3A). The sequence features of the AREs and GREs in these genes are summarized in Table 2. The resulting plasmids were individually electroporated into PF 29-13-Rluc and BF SM-Rluc cell lines, where Renilla luciferase is constitutively expressed from the β-tubulin locus and used to normalize for cell number. A construct containing the 3′UTR of *actin A* served as a control. Twenty-four hours after tet induction, equal numbers of cells were harvested for luciferase activity measurements and qRT-PCR. Compared to the *actin A* control, different 3′UTRs exerted distinct effects on reporter expression at both luciferase activity and RNA levels in PFs (Figure 3B) and BFs (Figure 3C). These results indicate that 3′UTRs harbor critical regulatory elements in *T. brucei* and play a key role in determining gene expression levels.

**TABLE 2:** Wild-type and mutated ARE or GRE sequence of selected genes.

Gene (gene ID)	3′UTR length (nt)	Motif (cluster)	Motif sequence^a^	Motif start location (nt)
KREPB6 (Tb927.3.3990)	319	GRE (V)	GUGCGUUUGGGUU	98
		GREm	GUGCG**CC**UGGGUU	
MCC-β (Tb927.11.6630)	827	ARE (III)	AUUUAUUUAUUUAUUUUUU	163
		AREm	AU**CC**A**CC**UAU**CC**AUUUUUU	
		GRE (V)	GUGUGUUUGUGGG	523
		GREm	GUGUG**CUC**GUGGG	
PK (Tb927.11.790)	717	ARE (V)	UAUUAUUUAAUA	257
		AREm	UAUUA**CC**UAAUA	
		GRE (V)	GGGUGUUUGUGUA	674
		GREm	GGGUG**CC**UGUGUA	
TOP2 (Tb927.9.5590)	738	GRE (IV)	GUUUGUUUGUUUUGU	596
		GREm	G**CC**UGU**CC**G**CC**UUGU	
ICP (Tb927.8.6450)	670	ARE (III)^b^	AUAUAUUUAUUUAUUUAUAUA	49
		GRE (III)	GUUUGUUUGUUUGUUUU	81
ICP (Tb427.08.6450)	679	ARE (Class III)^b^	AUAUAUAUAUAUAUAUAUAUAUAUAUA	49
		GRE (III)	GUUUGUUUGUUUGUUUU	91

*^a^*The repeats of pentamer AUUUA or GUUUG in each gene are underlined, and point mutations of “U” to “C” in the core pentamer are bolded. For reporter assay of ICP 3′UTR, we used ARE/GRE deletion instead of mutation due to the length of elements.

*^b^*The ARE sequence of ICP 3′ UTR of *T. brucei* TREU 927 listed in Tritrypdb (http://tritrypdb.org/tritrypdb/) contains AUUUA repeats. While corresponding location in Lister 427 strain is masked with “N.” Our sanger sequencing data showed that it contains only “AU” repeats, no AUUUA repeats, which belongs to class III ARE.

**FIGURE 4:**
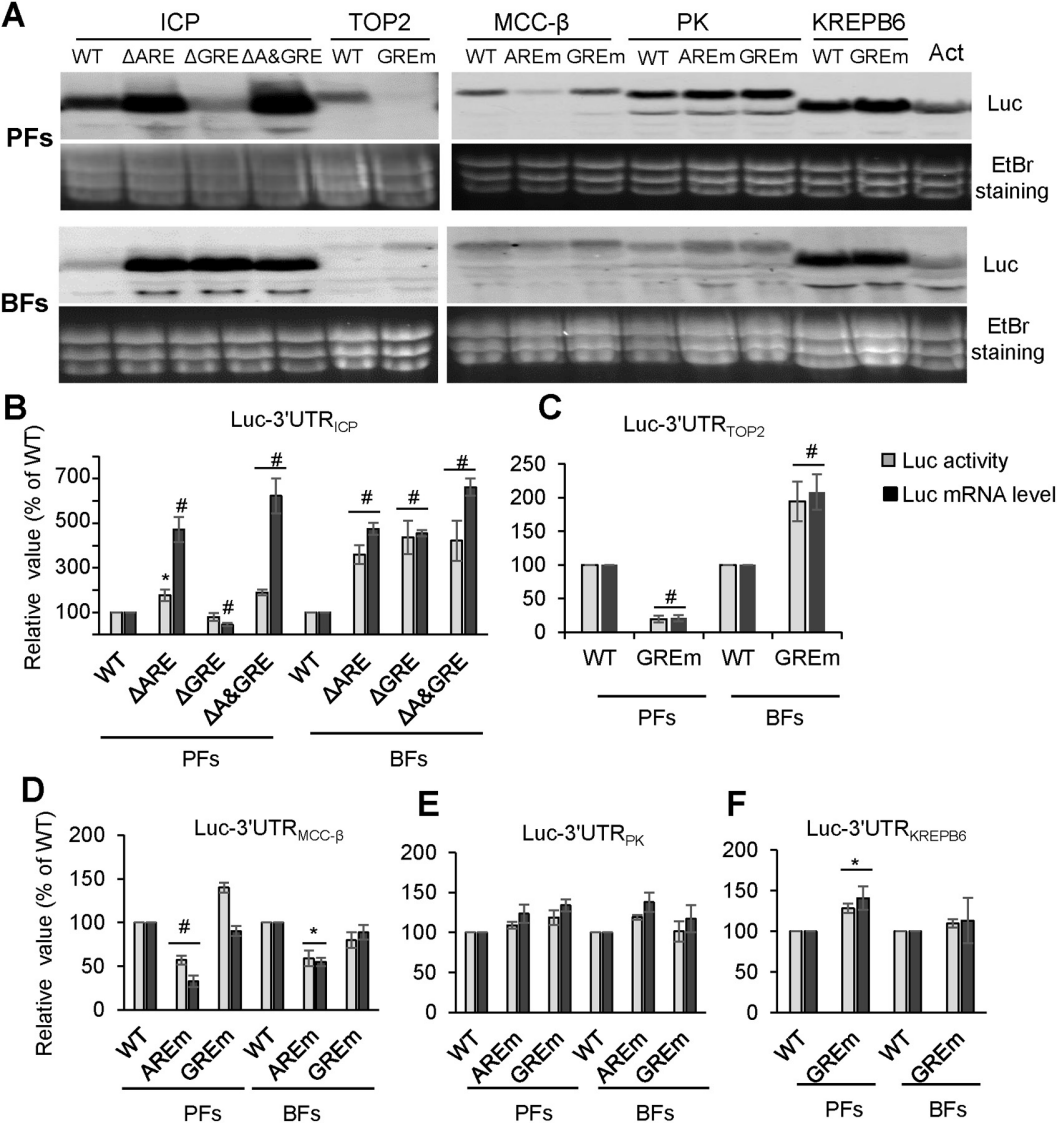
Effects of AREs and GREs on reporter gene expression. **(A)** Northern blot analysis of steady-state luciferase mRNA levels in PFs **(A)** and BFs **(B)** for reporters containing 3′UTRs with wild-type or mutant ARE/GRE elements. Hybridization was performed using a luciferase probe, and EtBr staining of rRNA served as a loading control. At least three independent experiments were performed for each of the three phleomycin-resistant populations. Data shown are from one representative population; similar results were obtained for the other populations. **(B–F)** Luciferase activity and mRNA levels of reporter constructs containing wild-type or mutant 3′UTRs from *ICP* **(B)**, *TOP2* **(C)**, *MCC*-β **(D)**, *PK* **(E)**, and *KREPB6* **(F)** in PFs and BFs. Values obtained from reporters with wild-type 3′UTRs were normalized to 100. WT, wild type; ΔARE, ARE deletion; ΔGRE, GRE deletion; ΔA/GRE, ARE and GRE dual deletion; AREm, ARE mutation; GREm, GRE mutation (see Table 2 for details). At least three independent experiments were performed for each of the three phleomycin-resistant populations. Error bars indicate the standard error of the mean. Statistical significance: ^#^*P* < 0.01, *P < 0.05 versus the corresponding wild-type construct.

The roles of AREs and GREs in regulating gene expression were also evaluated using reporter assays. Mutated 3′UTRs, in which AREs or GREs were altered (Table 2), were cloned into pLew79, and the resulting plasmids were introduced into PF 29-13-RLuc and BF SM-Rluc cell lines. After 24 h of the induction, equal numbers of cells were harvested for RNA isolation and luciferase activity measurements. Northern blot analysis revealed that mutation of the ARE, or combined mutation of both ARE and GRE in the 3′UTR of *ICP*, led to increased Fluc mRNA levels in both life stages of *T. brucei* (Figure 4A). In contrast, mutation of the GRE alone caused a marked reduction of Fluc mRNA in PFs but a significant increase in BFs (Figure 4A). These findings were further confirmed by luciferase activity measurements and real-time RT-PCR (Figure 4B). Together, the results indicate that the ARE in the 3′UTR of *ICP* acts as a potent destabilizing element at both life stages, whereas the GRE regulates gene expression in a life stage-specific manner, functioning as a stabilizing signal in PFs and a destabilizing signal in BFs; notably, the destabilizing activity of the ARE outweighs the regulatory influence of the GRE in PFs.

The regulatory roles of AREs and GREs were also observed in other tested 3′UTRs. Northern blot, qRT-PCR, and luciferase activity assays showed that mutation of the GRE in the *TOP2* 3′UTR downregulated reporter expression in PFs but upregulated it in BFs (Figures 4A,C), indicating a life stage-specific regulatory function similar to that of the GRE in *ICP*. In contrast, GRE mutations in the 3′UTRs of *MCC*-β, *PK*, and *KREPB6* had minimal effects in both life stages (Figures 4A,D–F). Mutation of the ARE in the *MCC*-β 3′UTR caused a marked reduction in reporter expression in both PFs and BFs (Figures 4A,D), suggesting that the ARE functions as a stabilizing element. Conversely, ARE mutation in the *PK* 3′UTR slightly increased reporter expression in both life stages (Figure 4E), consistent with a destabilizing role. Together, these results demonstrate that AREs and GREs are important regulatory elements in *T. brucei*, with gene-specific effects that can be strong or weak, and either dependent or independent of life stage.

### The GRE of TOP2 3′UTR differentially affects mRNA stability

**FIGURE 5:**
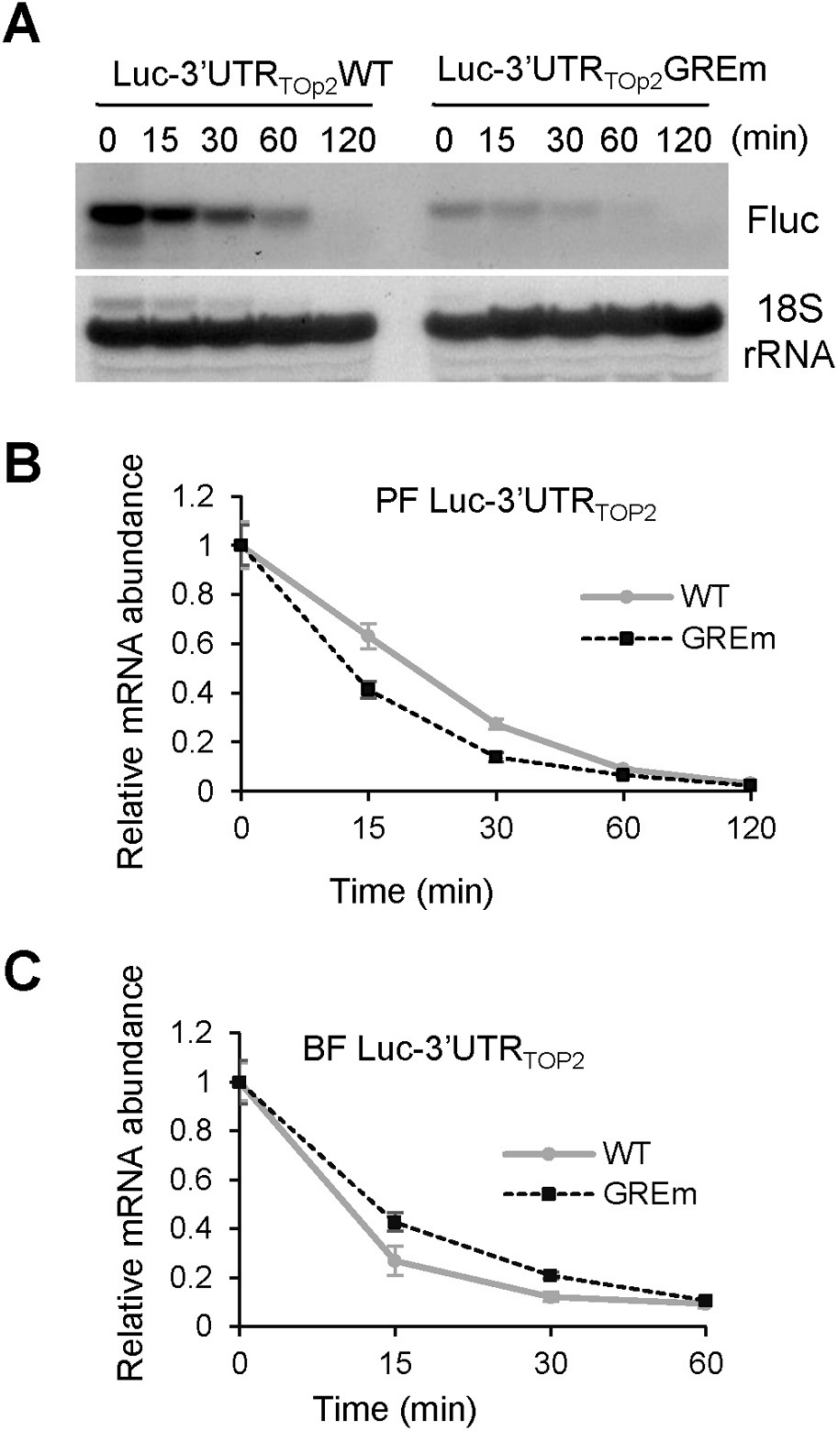
Effect of the GRE in the *TOP2* 3ffect of the GRE in thetability. **(A)** Northern blot analysis of luciferase transcripts containing wild-type or GRE-mutant *TOP2* 3′UTRs at indicated time points after actinomycin D treatment to inhibit transcription in PFs trypanosomes. Hybridization to 18S rRNA served as a loading control. Data are representative of a single replicate from one of the three phleomycin-resistant populations. **(B)** Quantification of Northern blot signals in **(A)**. Transcript levels at time 0 (immediately after actinomycin D addition) were set to 1. **(C)** qRT-PCR measurement of the half-lives of luciferase transcripts fused to wild-type or GRE-mutant *TOP2* 3′UTRs in BFs. Error bars indicate the standard error of the mean.

The effect of GRE of *TOP2* 3′UTR on mRNA stability was observed in both PF and BF *T. brucei.* After 24 h of tet induction of Fluc expression, PF and BF cells expressing either Luc-3′UTR_*TOP*2_WT or Luc-3′UTR_*TOP*2_GREm were treated with actinomycin D to block transcription, and total RNA was collected at the indicated time points. FLuc mRNA in PFs was analyzed by Northern blot, whereas in BFs it was quantified by qRT-PCR due to the very low abundance of Luc-3′UTR_*TOP2*_ transcripts. Compared to the WT GRE, mutation of the GRE accelerated Fluc mRNA degradation in PFs (Figures 5A,B) but slowed degradation in BFs (Figure 5C). These results indicate that the GRE of the *TOP2* 3′UTR differentially regulates reporter gene expression by modulating mRNA stability in opposite directions at different life stages of *T. brucei*.

### Identification of candidate proteins that bind to the GRE of *ICP* 3′UTR

**FIGURE 6:**
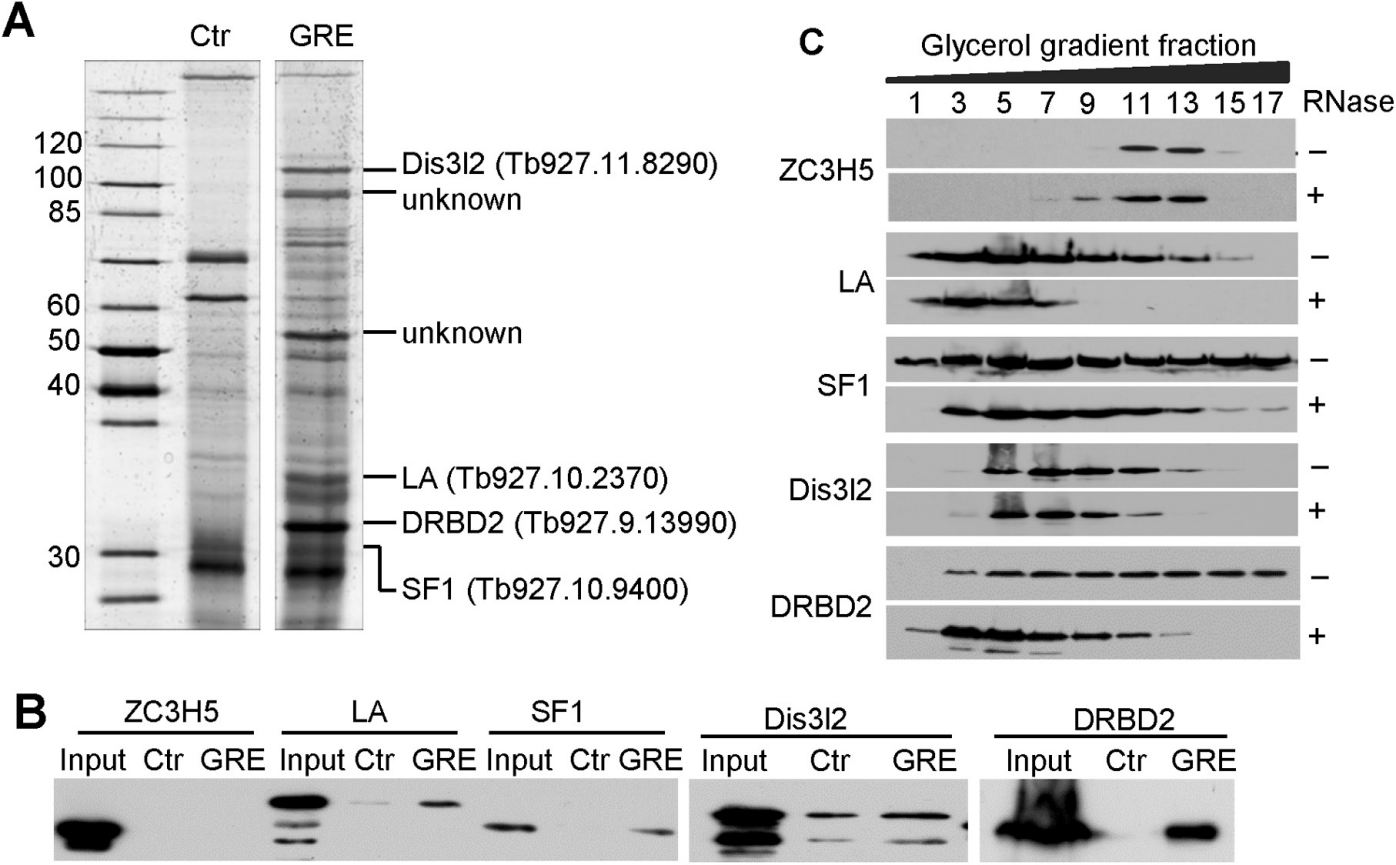
Screening for specific GRE-binding proteins. **(A)** Silver-stained SDS-PAGE of proteins pulled down by *ICP* GRE RNA. MS-identified proteins are indicated on the right. The leftmost lane shows protein markers, with molecular weights (kDa) indicated on the left. **(B)** RNA pull-down assay of candidate proteins. TAP-tagged candidates were ectopically expressed in PF 29-13 cells, and pull-down experiments were performed using cell lysates. Bound proteins were detected with a PAP antibody. Images are representative of three independent biological replicates. **(C)** Glycerol gradient ultracentrifugation of lysates from PF 29-13 cells expressing TAP-tagged candidate proteins, with or without RNase treatment. Data are representative of three independent biological replicates.

Given that no GRE-binding proteins have been characterized in *T. brucei*, we sought to identify candidate GRE-binding proteins using RNA pull-down experiments. A 40-nt GRE sequence corresponding to the GRE in the *ICP* 3′UTR (ICP GRE) was incubated with PF 427 cell lysates, while a 37-nt U-rich sequence served as a control. Prey proteins were separated by SDS-PAGE and visualized by silver staining. Bands unique to the ICP GRE lane were excised and analyzed by mass spectrometry (MS) (Figure 6A). Based on the MS data, four proteins, LA, SF1, DRBD2, and DIS3-like exonuclease 2 (Dis3l2), were selected for further RNA pull-down verification. ZC3H5, a putative RNA binding protein with CCCH-type zinc finger motif, which was not detected in the MS data, was included as a negative control.

PF cell lines expressing C-terminal TAP-tagged versions of these target proteins from the β-tubulin locus were generated, and RNA pull-downs were performed as described above in these cell lines, followed by western blot detection of TAP-tagged proteins. ZC3H5 showed no interaction with either ICP GRE or control RNA, whereas DRBD2, SF1, and LA displayed specific binding to ICP GRE RNA. Dis3l2 bound to both ICP GRE and control RNA with similar affinity (Figure 6B).

To determine whether these proteins associate with RNAs within larger complexes *in vivo*, glycerol gradient fractionation was performed with and without RNase A treatment. DRBD2 and LA shifted to lighter fractions after RNase treatment, whereas SF1, Dis3l2, and the control ZC3H5 showed minimal or weak changes in distribution (Figure 6C). These results indicate that DRBD2 and LA associate with RNAs in higher-order complexes *in vivo*. Collectively, DRBD2 and LA are potential GRE-binding proteins, and their association with RNA is likely mediated through recognition of specific GRE sequences.

### DRBD2 is involved in *ICP* gene expression regulation in PFs

**FIGURE 7:**
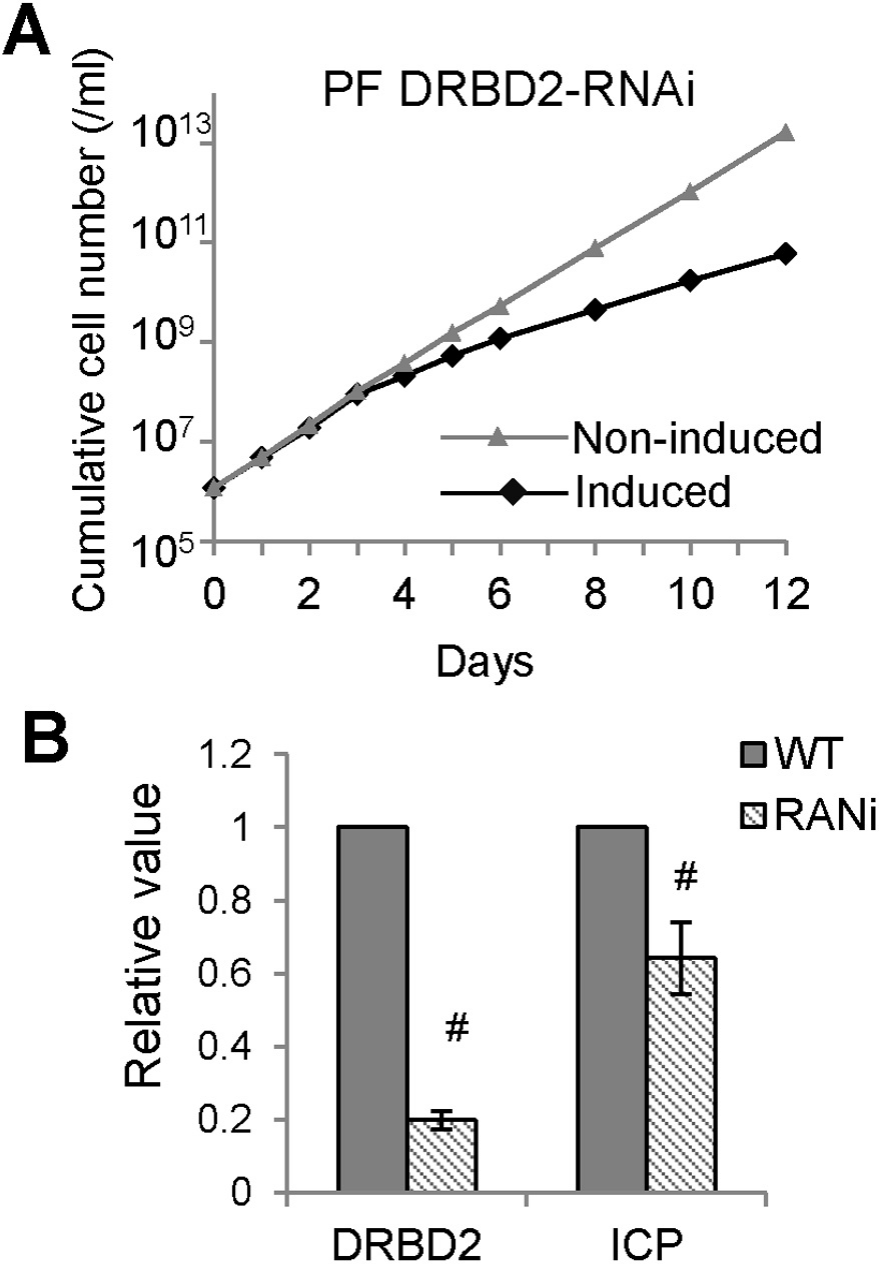
RNAi of DRBD2 reduces ICP expression. **(A)** Growth curve of the PF DRBD2-RNAi cell line. “Induced” indicates tetracycline treatment to trigger RNAi; “Non-induced” indicates cells without tetracycline. **(B)** qRT-PCR analysis of *DRBD2* and *ICP* mRNA levels in PF DRBD2-RNAi cells. *n* = 3 Statistical significance: ^#^*P* < 0.01.

Given that DRBD2 binds specifically to the GRE in the *ICP* 3′UTR *in vitro*, we next investigated whether DRBD2 depletion affects *ICP* expression. A tetracycline (tet)-inducible PF DRBD2-RNAi cell line was generated to assess the impact of DRBD2 knockdown on *ICP* expression in PFs. Upon tet-induced DRBD2 knockdown, cell growth was markedly inhibited, indicating that DRBD2 is essential for cell viability (Figure 7A). After 48 h of tet induction, total RNA was extracted and DRBD2 and ICP mRNA levels were quantified by qRT-PCR. Compared to uninduced control cells, DRBD2 and *ICP* transcript levels were reduced by approximately 80 and 30%, respectively (Figure 7B). These results indicate that DRBD2 is involved in regulating *ICP* expression in PFs of *T. brucei*. Given its specific binding to the GRE in the *ICP* 3′UTR, we speculate that DRBD2 modulates *ICP* expression through direct interaction with this GRE.

## Discussion

In this work, we identified two important regulatory elements, ARE and GRE, in *T. brucei* using published transcriptome data from various sources. These two elements are highly abundant in the 3′UTRs of *T. brucei* transcripts; up to 18.5 and 24% of genes with known 5′UTRs and 3′UTRs contain AREs and GREs, respectively (Table 1). In contrast, 7 and 5% of human genes contain AREs and GREs, respectively (Halees et al., 2011). AREs and GREs are enriched in the 3′UTRs of developmentally regulated mRNAs (Figure 1B). Nonetheless, the total number of regulated genes we analyzed was 327, a relatively small fraction of the total number of 5,840 genes. The datasets of regulated genes produced by different laboratories are quite different (Briggs et al., 2023; Jensen et al., 2009; Kolev et al., 2010; Naguleswaran et al., 2021; Nilsson et al., 2010; Siegel et al., 2010), and only the consensus genes were extracted and analyzed herein (Supplementary Table 1). The factors contributing to dataset uniformity include trypanosome strain, detection method, culture conditions, and bioinformatic tools used. Fadda et al. (2014) generated a list of 450 regulated genes that showed at least two-fold changes in expression in 3 of 4 sets of available RNA-seq data. Of these, 336 genes have defined 5′UTRs and 3′UTRs, and only 130 of these genes were present in the set of 327 genes analyzed herein. To exclude the possible effect of data source on regulatory element distribution, we analyzed the ARE and GRE content of the 336 developmentally regulated genes described in the study by Fadda et al. (2014); the results (Supplementary Figure 3) were very similar to those shown in Figure 1B.

We analyzed the regulatory role of AREs and GREs in the 3′UTRs of *ICP, TOP2*, *MCC*-β, *PK*, and *KREPB6* genes using reporter assays. The results showed that majority of the tested elements contribute to the regulation of reporter expression; the regulatory roles of these elements can be strong or weak and can enhance or reduce gene expression (Figure 4). These differences are not necessarily related to element length, number of core pentamer repeats, or life stage (Table 2). Mutation of the GRE within the *PK* or *KREPB6* 3′UTR resulted in weaker increase of reporter expression in both PFs and BFs (Figures 4A,E,F). GREs of *ICP* and *TOP2* 3′UTRs displayed different functions at the two life stages. Their mutations resulted in an obvious decrease in reporter mRNA and protein level in PFs and a significant increase in reporter mRNA and protein level in BFs (Figures 4A–C). This life stage-specific function is most likely achieved by changing mRNA stability differentially, as shown by the results of Luc-3′UTR_*TOP2*_ mRNA stability assays (Figures 5B,C). In contrast, the ARE of the *ICP*, *MCC*-β and *PK* genes function similarly at both life stages. Mutation of the ARE in the *MCC*-β 3′UTR reduced reporter expression (Figures 4A,D), whereas mutation of the AREs in the *ICP* and *PK* 3′UTRs increased reporter expression (Figures 4A,B,E). Therefore, both AREs and GREs are very important regulatory elements in *T. brucei*, and their regulatory roles are differential and gene specific.

AREs were originally classified into three classes based on the presence of AUUUA pentamers. Class I and class II contain AUUUA repeats in AU-rich regions; class III contains an AU-rich region without AUUUA and is difficult to screen from genome or transcriptome database based only on sequence or bioinformatic analysis (Barreau et al., 2005). In this work, AREs were classified into five clusters according to the number of AUUUA pentamer repeats (Table 1), and this classification has been adopted in previous studies (Halees et al., 2011; Khabar, 2017). The original class III ARE was not included; nonetheless, this does not mean that class III AREs are insignificant. Class III AREs have been shown to function as regulatory elements for a number of oncogenes and cytokines, including *c-jun*, *p53*, *GLUT1*, *hsp70*, and others (Deka and Saha, 2021; García-Mauriño et al., 2017; Haronikova et al., 2019; Makita et al., 2021; Vlasova and Bohjanen, 2008). The ARE of the *ICP* 3′UTR used in the reporter assay was amplified from *T. brucei* strain 427 and is a class III ARE with the sequence shown in Table 2. The *ICP* 3′UTR sequence used for computational analysis in this work is derived from strain 927; its ARE, which contains three overlapping AUUUA motifs, belongs to cluster III (Table 2). The reporter assay results indicated that mutation of the ARE in the *ICP* 3′UTR increased mRNA levels significantly in both PFs and BFs, suggesting an important regulatory role of class III AREs in *T. brucei*. Furthermore, De Gaudenzi et al. (2013) and Najafabadi et al. (2013) reported that AREs lacking AUUUA also represent a conserved element in some trypanosomatid transcripts; this result certainly widens the abundance and importance of AREs.

CELF1 is currently the only protein known to bind to GRE; it does so through its RRM domain, and it mediates the turnover of GRE-containing mRNAs in mammals (Qin et al., 2024). A BLAST search using the CUGBP1 protein sequence did not yield a significant trypanosomatid homolog. We reported here that DRBD2 binds to the GRE sequence of the *ICP* 3′UTR *in vitro* (Figures 6A,B). DRBD2, same as CELF1, is an RRM domain-containing protein. It is believed to be a homolog of yeast Gbp2, which mediates mRNA export and telomere length maintenance (Wippel et al., 2019). DRBD2 knockdown resulted in a reduction in the *ICP* mRNA level in *T. brucei* PFs (Figure 7B), consistent with the observed repression of Luc-3′UTR_*ICP*_ expression in PFs resulting from deletion of the GRE in the ICP 3′UTR (Figures 4A,C). Therefore, we speculate that DRBD2 regulates *ICP* expression in PFs through binding to its GRE. However, whether DRBD2 could down-regulate ICP expression in BFs in a manner similar to that caused by GRE deletion (Figures 4B,C) remains unknown due to our lack of success in generating an DRBD2 RNAi cell line in BFs. In future work, we will examine whether DRBD2 binds to the GRE through its RRM domain, identify novel GRE-binding proteins and characterize their interactions with GREs.

## Materials and methods

### Generation of AREs and GREs datasets

All *T. brucei* gene transcripts with annotated 5′ and 3′UTRs were extracted from RNA-seq data of two previous studies focusing on cultured life stages of *T. brucei* strain Lister427 (Jensen et al., 2009; Nilsson et al., 2010) and subsequently merged into a full-length transcript dataset (Supplementary Table 1). For genes exhibiting alternative 5′ splice sites or alternative 3′ polyadenylation sites, the longest 5′ and 3′UTR sequences (<5,000 bp) were selected. AREs and GREs were classified into five clusters based on the number of AUUUA and GUUUG repeats, respectively (Table 1). Using regular expressions of PERL programming language, we matched the AREs and GREs motif sequences as shown in Table 1 in the full-length-transcript dataset by using the similar algorithms as described previously (Halees et al., 2011). Briefly, ARE and GRE motif sequences were individually searched in the 5′UTR, coding sequences (CDS) and 3′UTR of each transcript. The longest sequence match was recognized as the best match and the corresponding gene was assigned to the cluster of longest matched motif. Only one mismatch was allowed while no insertion or deletion was allowed. For a transcript, if neither ARE nor GRE motif was found, the gene was considered non-GRE or non-ARE, respectively. Genes that displayed developmental changes in expression in both references (Jensen et al., 2009; Nilsson et al., 2010) at a cutoff of *P* < 0.05 were recognized as “truly” developmentally regulated genes; all other candidate genes were analyzed as non-developmentally regulated genes. The half-life of each candidate gene was extracted from reference (Fadda et al., 2014), in which mRNA decay rates in PF and BF *T. brucei* were measured by comparing RNA-seq data collected at various time points after inhibition of transcription. The gene details mentioned above are shown in Supplementary Table 1. Gene Ontology (GO) terms enrichment analysis of selected groups of genes was performed in PANTHER.^1^ Gene sets containing AREs and/or GREs were compared against the full set of 5,840 genes as the reference list, using the PANTHER GO-slim annotation. Statistical significance was assessed with Fisher’s exact test, for multiple test correction, false discovery rate (FDR) was calculated.

### Plasmid construction

To generate pHD1344-Rluc, the coding sequence of a *Renilla* luciferase gene (*Rluc*) was amplified by PCR and cloned into pHD1344-tub vector (Li et al., 2017) after digestion with *BamH*I and *Hind*III. pLew79 contains a firefly luciferase gene (*Fluc*) followed by an aldolase 3′UTR. An *Xho*I site was introduced downstream of the aldolase 3′UTR in plew79 via site-directed mutagenesis kit according to the manufacturer’s instructions (ThermoFisher). The 3′UTRs of selected genes containing wild-type (WT) or mutated ARE or GRE were digested with *BamH*I and *Xho*I and inserted into similarly digested pLew79 to replace the aldolase 3′UTR, and the generated plasmids were designated pLew79-Luc-3′UTR_*gene_name*_WT, pLew79-Luc-3′UTR_*gene_name*_AREm, and pLew79-Luc-3′UTR_*gene_name*_GREm, respectively. The 3′UTRs with ARE or GRE mutations were generated by inserting 3′UTR WT into PMD18-T vector (Takara) followed by site-directed mutagenesis. Full-length genes *DRBD2* (Tb927.9.13990), *Dis3l2* (Tb927.11.8290), *LA* (Tb927.10.2370) and *SF1* (Tb927.10.9400) were amplified by PCR with *T. brucei* 427 genomic DNA as template. After digestion with specific restriction enzymes, the PCR products were cloned into similarly digested PHD1344-TAP yielding pHD1344-DRBD2-TAP, pHD1344-Dis3l2-TAP, pHD1344-LA-TAP, pHD1344-SF1-TAP and pHD1344-ZC3H5-TAP. DRBD2 RNA interference (RNAi) DNA fragments were amplified by PCR with *T. brucei* 427 genomic DNA as template and cloned to pZJM vector (Li et al., 2017) and generate pZJM-DRBD2-RNAi. All generated constructs were confirmed by Sanger sequencing. The primers used are listed in Supplementary Table 2.

### Cell culture and cell line generation

*T. brucei* PF 29-13 and BF SM were cultured in SDM-79 medium and HMI-9 medium, respectively, supplemented with 10% fetal bovine serum and selection markers. pHD1344-Rluc was linearized by Not I and electroporated into PF 29-13 and BF SM. Following puromycin selection (1 μg/mL), stable cell lines derived from single clones were obtained and designated PF 29-13-Rluc and BF SM-Rluc, respectively. Rluc expression was evaluated using luciferase activity assays, and one representative clone from each life-cycle stage was selected for subsequent experiments. A series of pLew79-Luc-3′UTR plasmids containing different 3′UTRs with wild-type or mutated AREs or GREs were linearized by *Not*I and individually transfected into PF 29-13-Rluc and BF SM-Rluc. After selection with phleomycin (2.5 μg/mL), resistant populations were obtained from each transfection to avoid potential variability arising from rDNA integration events, and their expression of Luc in a tetracycline (tet)-dependent manner was measured. For each construct, three independent transfections were performed, yielding three resistant populations. To generate a tet-inducible RNAi cell line, PF 29-13 was transfected with *Not*I-linearized pZJM-DRBD2-RNAi. The resulting phleomycin-resistant clone was named PF *DRBD2*-RNAi. Three clones were evaluated by quantitative PCR to assess RNAi efficiency, and the clone exhibiting the strongest knockdown was selected. The PF cell lines expressing TAP-tagged DRBD2, Dis3l2, LA, SF1 and ZC3H5 were generated by transfecting *Not* I-linearized pHD1344-DRBD2-TAP, pHD1344-Dis3l2-TAP, pHD1344-LA-TAP, pHD1344-SF1-TAP and pHD1344-ZC3H5-TAP into PF 29-13, respectively, and selecting with puromycin. Expression of TAP-tagged proteins was determined by western blot, for each construct, the clone exhibiting the highest expression level was selected for further analyses.

### Luciferase reporter assay

Cell lysates were prepared using passive lysis buffer (Promega), and Rluc and Luc activities were measured using the Dual Luciferase Assay kit (Promega) according to the manufacturer’s instructions. For each resistant population or clone, at least three independent measurements were performed.

### RNA isolation and quantitative reverse transcript PCR (qRT-PCR)

10^7^ cells were lysed in 1 mL of TRIzol Reagent (Life Technologies), and total RNA was isolated according to the manufacturer’s instructions. The target mRNA levels were measured by qRT-PCR as described previously (Li et al., 2017). Briefly, quantification of RNA was performed using a NanoDrop 2000 spectrophotometer (Thermo). Samples with an OD_260/280_ ratio > 1.95 and a concentration above 1 μg/μL were used for downstream analyses. RNA integrity was further assessed by agarose gel electrophoresis followed by ethidium bromide (EtBr) staining of rRNA bands. Genomic DNA removal and reverse transcription were carried out using the PrimeScript™ RT reagent Kit with gDNA Eraser (Takara). qPCR was performed using a SYBR Green-based reagent (TB Green Premix Ex Taq, Takara). Control reactions lacking reverse transcriptase were included to exclude genomic DNA contamination. Target mRNA levels were normalized to β-tubulin mRNA, and relative gene expression was calculated using the ΔΔCt method. The sequences of the primers are listed in Supplementary Table 2.

### Northern blot

Northern blot was performed as described previously (Li et al., 2017). RNA samples from Luc-expressing cells were hybridized with a DIG-labeled probe specific to Luc. The rRNAs were observed through EtBr staining, which was used as a control. RNAs from PF Luc-3′UTR_TOP2_WT and PF Luc-3′UTR_TOP2_GREm cells treated with actinomycin D for the indicated times were hybridized with the labeled probe specific to *Luc*; the blots were then stripped and rehybridized with a probe specific to 18S rRNA, which was used as a control. Optic densities of the bands were quantified with NIH Image J.^2^ The sequences of the primers are listed in Supplementary Table 2.

### RNA pull-down and mass spectrometry analyses

RNA pull down was performed as described previously (Liu et al., 2019). A 40-mer GRE-containing RNA (5′-aggggauucugcuuuuuuguuuguuuguuuguuuuuguac-3′) and a control U-rich RNA oligo (5′-ggacauucauuuaauauuuuuucguua uauuuuuuug-3′) were synthesized and 5′ labeled with biotin. For each reaction, 400 nM RNA was incubated with 100 μL of washed streptavidin magnetic beads (Promega) at 4°C for 1 h. Depending on the experiment, 10^9^ PF cells were lysed with 1 ml of IPP150 buffer (150 mM NaCl, 10 mM Tris-Cl, pH 8.0, 0.1% NP40) supplemented with 1% Triton X-100, 100 μg of yeast tRNA, and 0.5 U/μL RNase inhibitor (Promega). Clear supernatant (500 μL) was added to the target and the control RNA-streptavidin beads for each binding reaction. After incubation at 4°C for 1 h, the beads were washed 6 times with IPP150 and then boiled in 1 × SDS loading buffer at 100°C for 10 min. The retrieved proteins were isolated on a 10% SDS-PAGE gel and silver-stained. Specific protein bands in gel slices were digested with trypsin and subjected to liquid chromatography-tandem mass spectrometry (LC-MS/MS) analysis using the service provided by the Beijing Genomics Institute (BGI, Shenzhen, China). The *T. brucei* protein database was downloaded from TriTrypDB.^3^

### Western blot

TAP-tagged proteins were detected by western blot as described previously. Briefly, protein samples were prepared from cells lysed directly with 1 × SDS loading buffer or from RNA pull-down complexes boiled with 1 × SDS loading buffer. TAP-tagged proteins were detected using a peroxidase conjugated anti-peroxidase (PAP) antibody (Sigma, 1:2,000) followed by chemiluminescence.

### RNase a treatment and glycerol gradient centrifugation

Approximately 1 × 10^9^ PF cells expressing TAP-tagged proteins were lysed in 2 ml of IPP150 buffer containing 1% Triton X-100 and EDTA-free protein inhibitor cocktail (Roche). The clear supernatant was divided into two portions; one portion was treated with RNase A (10 μg/mL) for 30 min at 4°C, and the other was mock-treated as a control. After centrifugation for 10 min at 4°C, the clear supernatant was subjected to 5–30% discontinuous glycerol gradient centrifugation at 38,000 rpm, 4°C for 8 h, with a SW40 Ti rotator (Beckman). Eighteen fractions were collected and used for western blot with PAP reagent.

### Statistical analysis

For analysis of the distribution of ARE- or GRE-containing genes in candidate genes and the half-life distribution of ARE- or GRE-containing genes, the hypergeometric test was used. Luciferase activity assays and northern blot band intensity quantifications were analyzed using one-way analysis of variance (ANOVA) for ICP, MCC-β, and PK, followed by the Holm-Bonferroni correction for multiple pairwise comparisons. For TOP2 and KREPB6, unpaired *t*-tests were used. All statistical analyses were conducted using Microsoft Excel and SPSS.

## Data Availability

The datasets presented in this study can be found in online repositories. The names of the repository/repositories and accession number(s) can be found in this article/Supplementary material.

## Footnotes

1
http://pantherdb.org/

2
https://imagej.net/NIH_Image

3
http://tritrypdb.org/tritrypdb/
