# Supplementary material for: Differential regulatory role of AU-rich and GU-rich elements in Trypanosoma brucei
Source: Front Microbiol. 2026 Jan 23;16:1724550. doi: 10.3389/fmicb.2025.1724550 (PMC12876127; doi:10.3389/fmicb.2025.1724550)
Supplement: Supplementary file 1 [file Data_Sheet_1.pdf]

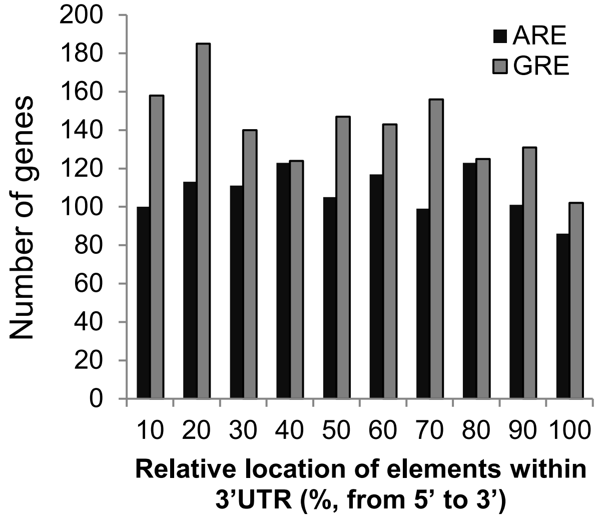

Fig. S1 Relative location of AREs and GREs within 3'UTRs

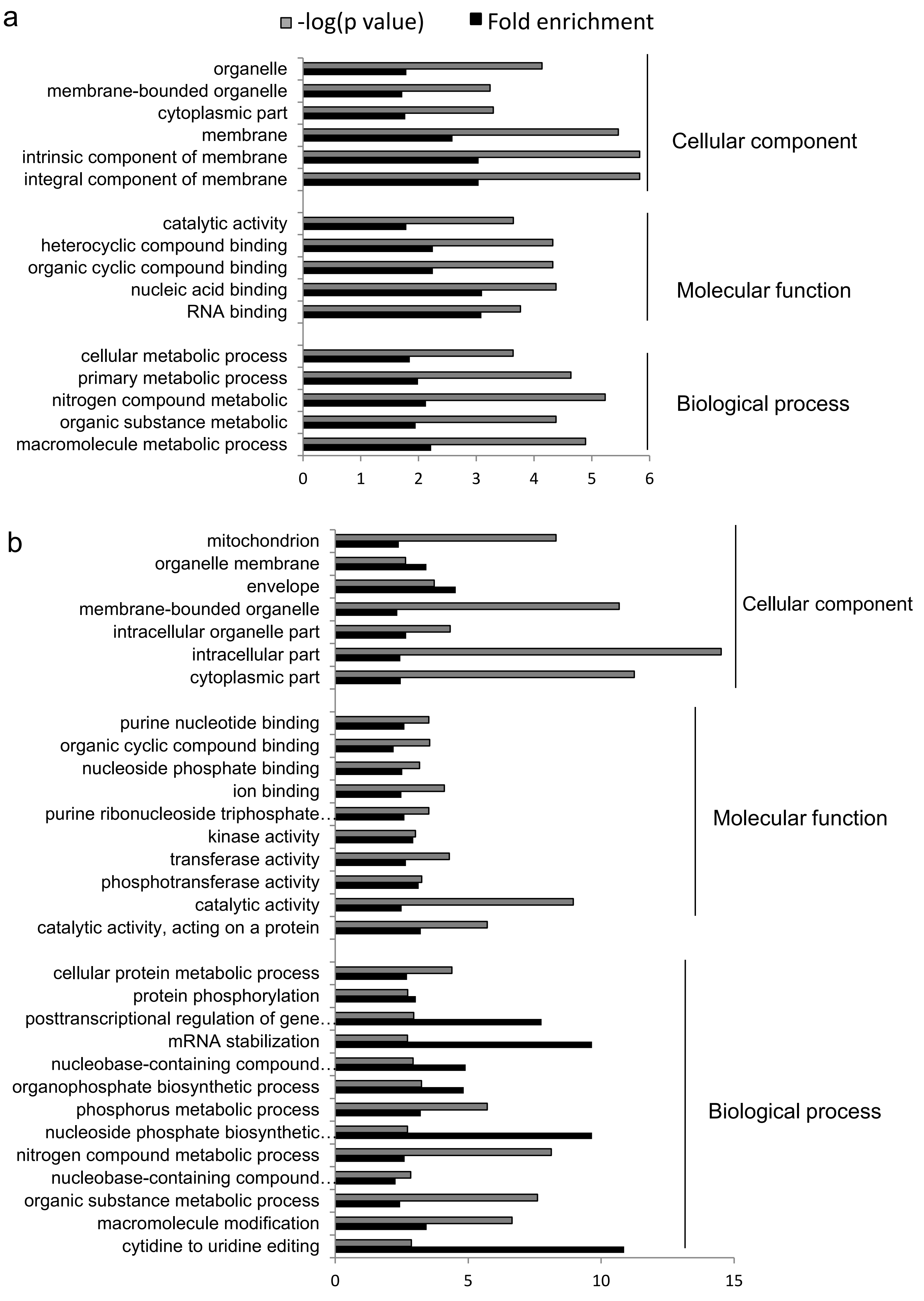
a
-log(p value)
Fold enrichment
organelle
membrane-bounded organelle
cytoplasmic part
membrane
intrinsic component of membrane
integral component of membrane
catalytic activity
heterocyclic compound binding
organic cyclic compound binding
nucleic acid binding
RNA binding
cellular metabolic process
primary metabolic process
nitrogen compound metabolic
organic substance metabolic
macromolecule metabolic process
Cellular component
Molecular function
Biological process
0
1
2
3
4
5
6
b
mitochondrion
organelle membrane
envelope
membrane-bounded organelle
intracellular organelle part
intracellular part
cytoplasmic part
purine nucleotide binding
organic cyclic compound binding
nucleoside phosphate binding
ion binding
purine ribonucleoside triphosphate…
kinase activity
transferase activity
phosphotransferase activity
catalytic activity
catalytic activity, acting on a protein
cellular protein metabolic process
protein phosphorylation
posttranscriptional regulation of gene…
mRNA stabilization
nucleobase-containing compound…
organophosphate biosynthetic process
phosphorus metabolic process
nucleoside phosphate biosynthetic…
nitrogen compound metabolic process
nucleobase-containing compound…
organic substance metabolic process
macromolecule modification
cytidine to uridine editing
Cellular component
Molecular function
Biological process
0
5
10
15

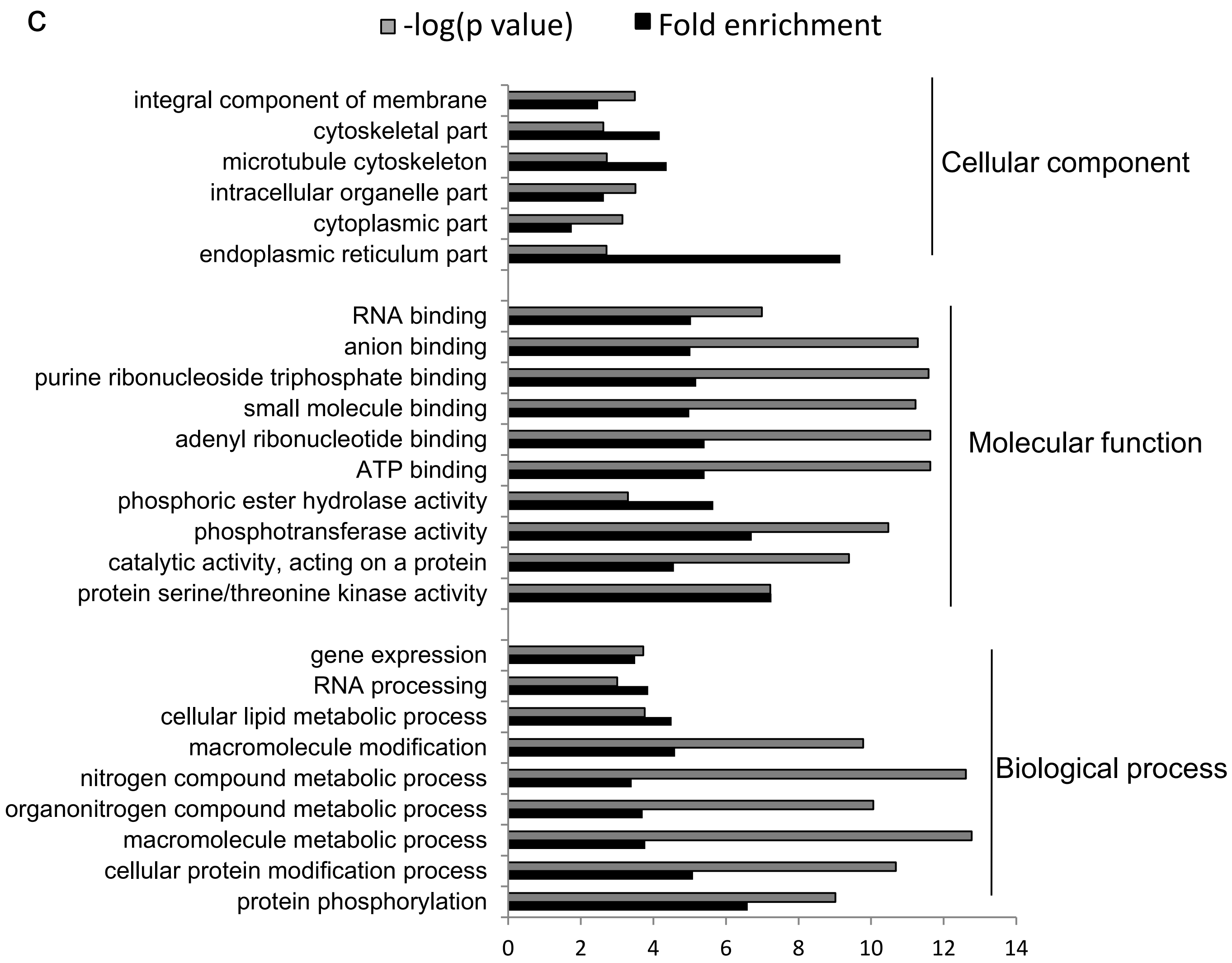

Fig. S2 Gene Ontology analysis of ARE-containing genes (a), GRE-containing genes (b) and ARE&GRE-containing genes (c).

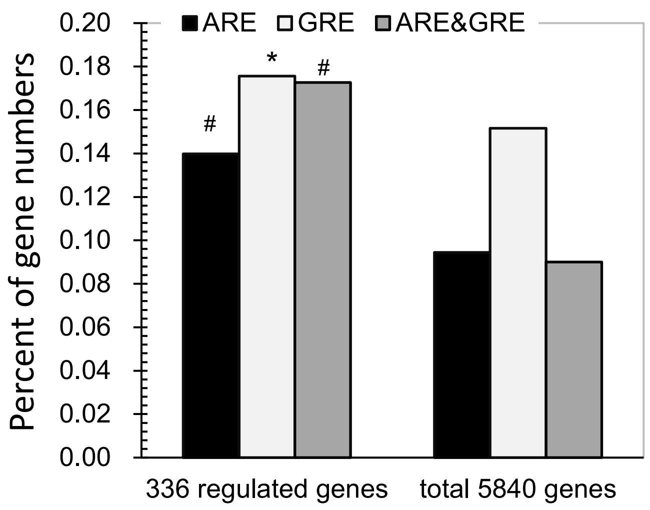

Fig. S3 AREs and GREs content in regulated genes
