# Supplementary material for: Differential regulatory role of AU-rich and GU-rich elements in Trypanosoma brucei
Source: Front Microbiol. 2026 Jan 23;16:1724550. doi: 10.3389/fmicb.2025.1724550 (PMC12876127; doi:10.3389/fmicb.2025.1724550)
Supplement: Supplementary file 3 [file Table_2.docx]

**Table S2.** Primers used in this study

| Name | Sequence (5'-3')^a^ | Target gene/usage |
| --- | --- | --- |
| renilucf | CACAAGCTTATGACTTCGAAAGTTTAT | *renilla* luciferase gene (*Rluc*) |
| renilucRv | CATGGATCCTTATTGTTCATTTTTGAG |  |
| plew79mutF | GAAGAAAATGGGGGGATCTCGAGCCCCAATTAAC | Introducing a *Xho*I site downstream of aldolase 3’UTR |
| plew79mutRv | GTTAATTGGGGCTCGAGATCCCCCCATTTTCTTC |  |
| I42UF | TGGATCCATAAGGCGGTATGTGGAA | ICP 3'UTR |
| I42URv | CCTCGAGAACAACAGAAGAAAGTAAAGC |  |
| top2 3' senP | GGATCCTCTAGATCGTTTCGAAAGTATATTC | TOP2 3'UTR |
| top2 3' antiP | GGTACCAGATCTCGCATGCACGTAGAACATATCAAG |  |
| pk 3' senP | GGATCCTCTAGAAGCGATGAGGCAAGTGGGTTCGTT | PK 3'UTR |
| pk 3' santiP | CTTAAGAGATCTAAAACAACGCATATTAGTGTCA |  |
| krep 3 senP | GGATCCTCTAGAGGTAAAAAACAAGCGAACA | KREPB6 3'UTR |
| krep 3 antiP | GGTACCAGATCTACGGTTTCAAAAGCGCGCATTCAC |  |
| CAA04803'F | GGATCCTCTAGAGGATTGATACCCGCACAGCAC | MCC-β 3'UTR |
| CAA04803'Rv | GGTACCAGATCTACCATCAACAGCCCACAA |  |
| act3'f | CACGGATCCTAACACCGGGTTGTGTGG | actin A 3'UTR |
| act3'rv | CACCTCGAGAATACTGCATAGATAACA |  |
| KRGREMF | gtttatatctgtgcgcctgggttttctgtg | KREPB6 3’UTR with GRE mutation (3’UTR_KREPB6_GREm) |
| KRGREMR | cacagaaaacccaggcgcacagatataaac |  |
| TOP2GREMF | gcctgtccgccttgtatttatgtgtgtgggt | TOP2 3’UTR with GRE mutation (3’UTR_TOP2_GREm) |
| TOP2GREMRv | gcccgtagatatgccttc |  |
| PKAREMF | ctgcagatattacctaatacagcgttttttttc | PK 3’UTR with ARE mutation (3’UTR_PK_AREm) |
| PKAREMRv | gaaaaaaaacgctgtattaggtaatatctgcag |  |
| PKAGMF | cccacgtgtgggtgcctgtgtatcctag | PK 3’UTR with GRE mutation (3’UTR_PK_GREm) |
| PKAGMRv | ctaggatacacaggcacccacacgtggg |  |
| CAAREMUTF | ggtttatgattatccacctatccattttttgcattggcgcagcac | MCC-β with ARE mutation (3’UTR_MCC-β_AREm) |
| CAAREMUTRv | caacgagacccatacaca |  |
| CAAGREMUTF | gaccggtgtgtgctcgtgggcactgattgc | MCC-β with GRE mutation (3’UTR_MCC-β_GREm) |
| CAAGREMUTRv | caaaccccaccaaagggttc |  |
| I42nDelAreF | ggattctgctttttttgtacag | ICP 3’UTR with ARE deletion (3’UTR_ICP_ΔARE) |
| I42nDelAreRv | ccttccttctccacttcca |  |
| I42DGREF | tttgtacagatgtataatcg | ICP 3’UTR with GRE deletion (3’UTR_ICP_ΔGRE) |
| I42DGRERv | aaAAGCAGAATCCTATA |  |
| I42DelAre2F | GGATTCTGCTTTTTTGTTTG | Pair with I42nDelAreRv to generate 3’UTR_ICP_ΔA&GRE |
| drbd2-f | CACCTCGAGATGCAAGGAGGAAATATGTTC | *DRBD2* |
| drbd2-rv | CACGGATCCTGAAGTGGATTTATTGTCAA |  |
| TbSF1F | CACAAGCTTATGGGGGAGAACCGTCGA | *SF1* |
| TbSF1R | CACAGATCTCAACTCGTCCAGAAACTCCTG |  |
| TbLAf | CACGTCGACATGCCACTTTCCTCCGAG | LA |
| TbLAr | CACAGATCTTTCACGTGACCGCTTGTG |  |
| TBrrp44f | CACGTCGACATGGATGAAACACCGAAT | Dis3l2 |
| Tbrrp44R | CACGGATCCAACGAGACTTGTGGGTAT |  |
| ZC3H5 TAP Fw | CACCTCGAGCCACC ATGATGCAAC GGGATACC | ZC3H5 |
| ZC3H5 TAP Rev | CACGGATCC ATGTAACC CAGTTTGTAG |  |
| RNAitDRDB2F | CACAAGCTTCGACGAGCATTCATCTGC | DRBD2 RNAi fragment clone |
| RNAitDRDEB2Rv | CACCTCGAGGAATTCCCATCATCGGGT |  |
| rRNA18sf | ATG AAG GAG GGT AGT TCG GG | 18S rRNA/northern blot |
| rRNA18sRV | CTT CCT CTA TTG AAG CAA TA |  |
| Luc probe f | CATAGAACTGCCTGCGTCA | Fluc/northern blot |
| Luc probe R | AAGACCTTTCGGTACTTCGTCC |  |
| Qluc-2F | TTGTTTTGGAGCACGGAAAGAC | Fluc/real-time PCR |
| Qluc-2Rv | AAGACCTTTCGGTACTTCGTCC |  |
| qMccB-Fw | AGGTGGGACGTATTACCCGA | MCC-β/real-time PCR, TAP and RT-PCR |
| qMccB-Rv | TCGCTATTTGGGCGATACCC |  |
| QKERPB6-Fw | GGAGTGTGATAGCGTTGCCT | KREPB6/real-time PCR QPCR primers |
| QKREPB6-Rv | TTCGCCAATTCCTGACCACA |  |
| QPK-Fw | GCAGTGGAGGATACAGCGTT | PK/real-time PCR |
| QPK-Rv | CTCCAACCCGCCGATACTAC |  |
| qTOP2Fw | TCGGTTCTCGTCAGCAACTC | TOP2/real-time PCR |
| qTOP2Rv | TTCGACGCAAAACCGAAACC |  |
| ICPQ2-F | TACACGTGGCTTCGTTCAGG | ICP/real-time PCR |
| ICPQ2-R | CGAGTGAAAGCGTGTGCTTC |  |
| qDRBD2-F | CGAAGTCGGTCGCAAATTGT | DRBD2/real-time PCR |
| qDRBD2-R | AATCTCAGCGCGTTCCACTT |  |
| b-tub Q-PCR Fw | TTCCGCACCCTGAAACTGA | β-tubulin/real-time PCR |
| b-tub Q-PCR Rev | TGACGCCGGACACAACAG |  |

^a^ Restriction sites are underlined.
